# Seagrass Meadows as a Foundational Concept for One Health

**DOI:** 10.1093/biosci/biaf208

**Published:** 2026-01-08

**Authors:** Richard K F Unsworth, Iestyn Comey, Benjamin L H Jones

**Affiliations:** Project Seagrass, Bridgend, CF31 2AQ, UK; Seagrass Ecosystem Research Group, Department of Biosciences, Swansea University, Swansea, SA28PP, UK; Project Seagrass, Bridgend, CF31 2AQ, UK; Project Seagrass, Bridgend, CF31 2AQ, UK

**Keywords:** marine biology, natural resources, plant biology, plant–animal interactions

## Abstract

Seagrass meadows are vital coastal ecosystems that embody the One Health paradigm, connecting human, animal, and environmental well-being. These highly productive habitats offer critical ecosystem services: They store carbon, stabilize shorelines, and filter pollutants and pathogens, bolstering climate resilience and water quality. As foundation species, seagrasses support diverse marine life and underpin global fisheries that provide nutrition, livelihoods, and cultural sustenance for coastal communities. Microbes enhance this function by actively reducing pathogens and detoxifying sediments, showcasing how microscale processes support broader societal health. However, anthropogenic pressures, especially nutrient pollution, are driving global decline. Case studies demonstrate that coordinated management and restoration can reverse this degradation and enhance ecosystem and social benefits. Integrating seagrass conservation into One Health frameworks highlights the need for proactive, multisectoral approaches. Protecting and restoring these meadows is a critical investment in resilient coastal communities, sustainable fisheries, and the overall health of human and planetary systems.

The concept of One Health represents a fundamental shift in how we approach global health challenges. Defined as a collaborative, multisectoral, and transdisciplinary approach, it operates across local, regional, national, and global scales (Pitt and Gunn 2024). At its core, its objective is to achieve optimal health outcomes by recognizing the profound interconnection among people, animals, plants, and their shared environment (OHHLEP et al. 2022).

The One Health approach is gaining momentum as an effective way to combat health threats at the human–animal–environment interface, particularly zoonotic diseases and antimicrobial resistance. Beyond infectious diseases, the framework addresses a wide range of interconnected issues, including food safety and security, environmental contamination, and climate change. The foundations of One Health are the principles of communication, coordination, and collaboration among diverse experts, including human health professionals, veterinarians, ecologists, and wildlife experts. By fostering this collaborative synergy, the framework seeks to protect global health security, improve food safety, and safeguard biodiversity and conservation (OHHLEP et al. 2022).

Within this holistic framework, seagrasses emerge as a model and an overlooked example of the One Health paradigm in action. These marine flowering plants form extensive underwater meadows worldwide, ranging from tropical to temperate coastal waters (Unsworth et al. 2022). Functioning as powerful ecosystem engineers, seagrasses modify their environment in ways that generate a cascading series of benefits, acting as both a foundation for diverse life and a critical sentinel of coastal health (Orth et al. 2006). The health of these meadows is directly tied to the well-being of the communities, economies, and ecosystems that depend on them (Cullen-Unsworth et al. 2014).

In the present article, we provide an account of why seagrass ecosystems are a foundational concept for One Health. We demonstrate this by bridging the scales from microbial symbiotic processes to macrolevel societal and policy contexts. Our analysis reveals that the health of seagrass meadows, the microbial communities they harbor, the animals they sustain, and the people who depend on them are all integral to a single, interconnected system, underscoring the urgent need for transdisciplinary conservation efforts.

## The seagrass ecosystem and its services

Seagrass meadows are among the most productive and diverse ecosystems on Earth, providing a suite of services that are indispensable to both the marine environment and human populations (Nordlund et al. 2016). These services are integral to the One Health framework, because they directly link the health of the environment to the health of animal and human populations. The critical functions of seagrass meadows can be categorized into four major types of ecosystem services: regulating, supporting, provisioning, and cultural services (Nordlund et al. 2016).

### Regulating services

Seagrass meadows provide a direct link to the One Health framework by mitigating climate change, a key threat to global health (Franchini and Mannucci 2015). As a powerful blue carbon storage system, seagrasses absorb, trap, and store carbon in marine sediments, a critical step in combating climate change and its cascading effects on human and animal well-being. Although they cover less than 1% of the ocean, they are responsible for an estimated 10% of the annual carbon sequestration in the ocean, trapping carbon in oxygen-depleted seabed sediments, where it can remain for centuries (Fourqurean et al. 2012).

The environmental regulation provided by seagrass also has a direct and immediate impact on human health and safety through its role in coastal protection (Forrester et al. 2024). The extensive root and rhizome systems of seagrasses stabilize the seabed and protect against erosion. In many cases, their dense leaf canopy acts as a natural baffle, dissipating up to 40% of wave energy (Fonseca and Cahalan 1992) and thereby reducing flooding and safeguarding coastal communities from extreme weather events. This protection is a direct One Health benefit, because it ensures the safety and economic security of people living in vulnerable coastal areas (Elnaiem et al. 2023, Quinn et al. 2023). An indirect benefit of reduced coastal flooding, potentially brought about by seagrass, is the reduction in human health concerns from waterborne pathogens and other concerns related to flooding (Quinn et al. 2023).

Furthermore, seagrasses act as natural filters, helping regulate water quality, a process essential for the well-being of people and animals. They trap suspended particles, sediments, and pollutants from surface runoff, including harmful chemicals (Short and Short 1984, Prystay et al. 2023), and filter out potential diseases and harmful microbes from the water column (Lamb et al. 2017). Increasingly, they are known for accumulating microplastics too (Li et al. 2023). In contrast to this filtration role, seagrasses may have a poorly understood role in influencing air quality via production of nitrous oxides and biogenic volatile organic compounds, such as isoprene and dimethyl sulfide. Recent evidence from the Mediterranean indicates that seagrass may be a significant input into regional biogenic volatile organic compound budgets and therefore have a significant influence on One Health (Saunier et al. 2025b). Dimethyl sulfide, in particular, is an important sulfur-containing compound that can then be released into the atmosphere, where it plays a key role in cloud formation and the atmospheric sulfur cycle, indirectly influencing air quality and climate regulation.

### Supporting services

Seagrasses, a diverse group of over 70 species of flowering plants, form the foundation of submerged grassland ecosystems in coastal waters spanning the globe, from the tropics to high latitudes (Short et al. 2007).

As foundation species and ecosystem engineers, seagrass meadows create a three-dimensional structure that provides critical food, shelter, and nursery habitats for a vast array of associated fauna, including commercially important fish and invertebrates, sea turtles, dugongs, and manatees (Hughes et al. 2009). The biodiversity within seagrass meadows is characteristically higher than in adjacent unvegetated areas, with the physical structure of seagrass providing protection from predators and creating the basis for complex food webs (Orth 1992, Henderson et al. 2017). As primary producers, foundation species help accumulate marine soil sediment, which effectively forms a home on which most of the biodiversity present in these systems is dependent.

### Provisioning and cultural services

Seagrass meadows function as critical nursery grounds, offering refuge and foraging opportunities for the juvenile stages of many commercially and recreationally important finfish and shellfish (Unsworth et al. 2018b). For some species, successful reproduction is contingent on access to seagrass (Hughes et al. 2009). Because the health and ultimately abundance of numerous fish populations underpins human food security and livelihoods (Willett 2005), seagrass represents a foundational environmental asset with direct implications for both animal and human health.

Seagrass ecosystems deliver direct, tangible, provisioning benefits to human health and well-being. Seagrass fisheries generate essential income for many coastal communities and underpin some of the world’s largest and most productive fisheries (Unsworth et al. 2018b). The condition of these fishing grounds sustains nutrient-rich diets for millions of people, directly addressing a core tenet of One Health: food security (Unsworth et al. 2014).

Beyond nutrition, seagrass provides cultural services that contribute to human physical and mental well-being. These include opportunities for recreational activities such as snorkelling, diving, and fishing. For some communities, seagrass meadows are also deeply embedded in cultural practices and traditions, enriching community identity and the quality of life. The value of these services reinforces the principle that a healthy environment is a prerequisite for healthy human societies. The comprehensive and diverse suite of services provided by seagrass meadows illustrates their fundamental role as a nexus within the One Health paradigm (see table 1).

**Table 1.** Links between seagrass ecosystem service provision and the One Health pillars.

Ecosystem service		Explanation of link to One Health	Percentage of species and bioregions service is present (those assessed)	Percentage of species and bioregions for which no data was available	One Health pillars (human, animal, environmental)	Reference
Regulating	Carbon sequestration and storage	Seagrasses store substantial blue carbon in sediment, mitigating climate change and reducing associated risks to human and animal well-being, directly supporting planetary health within the One Health framework.	100	34	Environmental (climate change mitigation)	(Fourqurean et al. 2012)
	Coastal protection	Seagrass beds dampen wave energy and stabilize sediments, reducing erosion and flooding risk, protecting coastal communities and economic security while maintaining habitat structure for marine species.	81	71	Environmental and human (coastal community safety, economic security)	(Forrester et al. 2024)
	Nutrient cycling and particle filtration	Seagrasses cycle nutrients and filter sediments, pollutants, microbes and microplastics, improving water quality and limiting pathogen exposure, thereby supporting healthier marine ecosystems.	100	16	Environmental (stability)human and animal (zoonotic pathogens)	(Tasdemir et al. 2024)
	Disease regulation and waterborne pathogen mitigation.	Seagrass meadows help regulate disease by reducing pathogen loads in coastal waters. Their ability to trap particles and microbes lowers the presence of harmful bacteria, parasites, and potential zoonotic pathogens. By improving water quality, seagrasses support healthier marine wildlife and reduce disease risks for coastal communities. Data on pathogen removal is currently limited to a handful of small case studies.	Limited data	n/a	Animal and human (disease and pathogen mitigation)	(Lamb et al. 2017, Tasdemir et al. 2024)
	Air quality	Seagrasses may influence air quality by contributing significant amounts of regional biogenic volatile organic compounds, with potential impacts on One Health.	Limited data	n/a	Human (health)	(Saunier et al. 2025a)
Supporting	Habitat creation	Seagrass meadows create structured habitats that support diverse marine species, sustaining food webs and fisheries that underpin ecosystem stability, wildlife health and human nutrition security.	100	13	Animal (biodiversity and conservation)	(Hughes et al. 2009)
	Nursery habitat provision	Seagrass meadows function as critical nursery grounds by enhancing the survival of juvenile fish and invertebrates. Their high productivity and sheltered conditions provide abundant food resources and reduce predation risk, increasing the number of individuals that reach maturity. These nursery functions directly support fisheries productivity and contribute to long-term population stability.	100	29	Animal and human	(Unsworth et al. 2018b)
	Soil (marine sediment) formation	Seagrasses trap and stabilize sediments, improving water clarity and creating habitats for benthic species, thereby benefiting both environmental and human health.	100	39	Animal and environment	(Maxwell et al. 2017)
	Biodiversity	Seagrass meadows support high species diversity, maintain complex food webs, and provide critical habitat, enhancing ecosystem resilience and safeguarding wildlife and ecosystem health.	100	29	Animal (species and habitat conservation)environment (resilience)	(Duffy et al. 2022)
Provisioning	Support for commercial and subsistence fisheries	Seagrass meadows provide critical nursery and feeding habitats for commercially important species, sustaining fisheries that support local livelihoods, food security, and economic well-being, linking marine ecosystem health to human health outcomes.	100	29	Human (food security, livelihoods, economic income)	(Unsworth et al. 2018b)
	Source of materials for human use	Seagrass has long provided raw materials for human use, including bedding and thatching, supporting cultural practices, sustainable livelihoods, and community resilience, linking environmental resources to human well-being.	41	55	Human (raw materials for crafts, insulation)	(Nordlund et al. 2018)
Cultural	Recreation (snorkelling, fishing, diving)	Healthy seagrass meadows support tourism and recreational activities that enhance mental and physical well-being while generating economic benefits for coastal communities.	100	21	Human (mental and physical well-being, tourism)	(Cullen-Unsworth et al. 2014)
	Cultural heritage	Seagrass has shaped human cultures for millennia, providing food, materials, and traditional practices that sustain community identity, knowledge systems, and cultural continuity, linking environmental stewardship to human well-being.	100	63	Human (community identity, traditional practices)	(Foster et al. 2025)

*Note:* The presence of these services across species and bioregions is assessed following data from (Nordlund et al. 2016).

## The human interface: Seagrass as a social–ecological system

Applying a One Health lense to seagrass meadows provides a unique and powerful framework for understanding the deep interconnections of human, animal, and environmental health. This perspective recognizes that the well-being of coastal communities and marine life is inseparable from the ecological condition of these ecosystems. Although the specific ways seagrass contributes to human well-being vary across geographies, environmental settings, and cultural contexts (de la Torre-Castro and Rönnbäck 2004, Cullen-Unsworth et al. 2014, McKenzie et al. 2021), a consistent pattern emerges: Where people live alongside extensive seagrass, their livelihoods, nutrition, and social fabric are shaped, directly or indirectly, by its presence. In many regions, this connection is as fundamental as daily food provision, buffering communities from the nutritional and emotional stress associated with uncertain or insufficient access to marine resources.

Seagrass meadows support poverty alleviation and community well-being, aligning closely with the One Health framework, which recognizes the interdependence of human, ecological, and animal health. By providing accessible fishing grounds requiring limited fishing capital and investment, they offer reliable sources of food and income, particularly for households with few livelihood alternatives (Jones et al. 2022). This accessibility not only supports nutritional health but buffers households against economic or climate shocks, reducing vulnerability and enhancing social resilience (Quiros et al. 2018).

Ecologically, seagrass habitats maintain water quality, stabilize sediments, and support diverse fish and invertebrate populations. By facilitating healthy ecosystems, they underpin sustainable fisheries, reduce fish disease, and enhance the safety and nutritional quality of seafood for human consumption. Their cultural and social values further promote mental well-being, identity and community cohesion through shared fishing practices, and stewardship traditions. Community-led restoration and monitoring initiatives reinforce these benefits, generating local employment, strengthening governance, and building environmental care. Therefore, seagrass meadows exemplify One Health by linking ecosystem integrity, sustainable livelihoods, food security, and social well-being.

## The wildlife interface

Approximately 75% of all emerging human infectious diseases originate in animals (Fong 2017), with a large proportion coming from wildlife. Practices such as habitat fragmentation, deforestation, the wildlife trade, and human encroachment are known to increase the frequency of contact at the wildlife–human–livestock interface (Jones et al. 2008). Seagrass meadows have associations with many species of megafauna, such as sharks, rays, dolphins, and turtles (Sievers et al. 2019), with increasing recognition of their role in supporting bird species (Unsworth and Butterworth 2021) and potentially being sites of foraging for larger terrestrial mammals (Hessing-Lewis et al. 2018). Although we lack direct evidence on how changes in seagrass extent or health affect disease spread, grazing waterfowl preferentially feed on seagrass rather than on terrestrial crops (Buchsbaum and Valiela 1987, Deans 1979), potentially reducing interactions with domesticated animals and limiting avian influenza transmission. Many seagrass-foraging bird species are known carriers of these viruses (APHA 2025). The loss of seagrass and shifting baselines have altered conservation efforts toward modifying farmland to attract historically seagrass-grazing wildfowl (Vickery et al. 1994), potentially further exacerbating wildlife–livestock interactions. Additionally, as natural filters of land-based contaminants, healthy seagrass meadows can reduce the prevalence and spread of infectious diseases via wildlife vectors.

## The microbial interface: Seagrass as a holobiont

Seagrass traps particles through its dense leaves and complex root–rhizome network, thereby slowing water flow and promoting sedimentation. The leaves capture suspended particles, whereas the roots stabilize and bind sediments, reducing resuspension. This process enhances water clarity, supports nutrient cycling, and creates stable habitats for diverse marine life (Maxwell et al. 2017). One type of particle in the water column that are trapped by seagrass are pathogens (Deng et al. 2021).

To fully appreciate the role of seagrass in One Health, it is essential to move beyond the plant itself and consider the complex, symbiotic relationships it forms with its associated microbial communities. This integrated biological unit, the plant and its microbiome, is known as a *holobiont* (Tarquinio et al. 2019). This perspective reframes the plant from a single entity to a complex superorganism that collectively responds to environmental changes (Ugarelli et al. 2017). The composition of the seagrass-associated microbial community varies with the host species and environmental conditions, indicating that these communities are dynamic and respond rapidly to disturbances (Conte et al. 2021). This intimate relationship means that the health of the seagrass plant is inextricably linked to the health of its microbiome.

The symbiotic relationship between seagrass and its associated microbes provides a powerful mechanism for mediating health at a microscopic scale, with macrolevel consequences. The most significant finding in this domain is the ability of healthy seagrass ecosystems to reduce the load of waterborne pathogens actively (Tasdemir et al. 2024). A pivotal study demonstrated a 50% reduction in the relative abundance of potential bacterial pathogens, including those dangerous to humans and marine animals, in the presence of seagrass meadows (Lamb et al. 2017). This remarkable sanitation effect is probably driven by the microbial communities living on the surface of the seagrass leaves. Research has shown that these epiphytic bacteria possess strong, broad-spectrum antibiotic activity against a wide range of pathogens, including human fecal bacteria and *Vibrio* species (Tasdemir et al. 2024).

The importance of this function extends beyond the seagrass meadow itself. Field surveys of over 8000 reef-building corals located adjacent to seagrass meadows revealed a twofold reduction in disease levels compared with corals at paired sites without adjacent seagrass (Lamb et al. 2017). This illustrates a direct, cascading benefit from a microscale process to the health of an entirely different ecosystem. The microbial health of the seagrass holobiont measurably translates to the health of other marine organisms, creating a demonstrably seascape-scale safer environment (Preston et al. 2025). The chain of events from a symbiotic biochemical function (microbial antibiotic production) to an ecological outcome (pathogen reduction) and finally to a societal benefit (reduced disease in marine life and safer coastal waters) we believe to be a perfect illustration of the One Health principle.

Seagrass meadows act as powerful nitrogen biofilters by integrating plant and microbial processes (Short and Short 1984, Aoki et al. 2020). Seagrass blades and roots take up dissolved inorganic nitrogen directly, and their dense belowground biomass traps organic matter and creates conditions that support diverse microbial communities (Ugarelli et al. 2017). Within anoxic microsites in the sediments, nitrifying and denitrifying bacteria convert reactive nitrogen into inert dinitrogen gas, permanently removing it from coastal waters (Zhou et al. 2021). This microbially mediated nitrogen cycling prevents excessive nutrient accumulation that otherwise drives eutrophication, harmful algal blooms, and hypoxia. By stabilizing water quality, seagrass meadows reduce toxin exposure risks for humans, protect fish and shellfish health, and support culturally and economically important coastal fisheries (Lamb et al. 2017). These functions align closely with One Health principles, demonstrating how microbial ecosystem processes sustain the interconnected well-being of humans, animals, and the environment. Protecting and restoring seagrass, therefore, strengthens public health, food safety, and climate resilience by maintaining the microbial services that underpin coastal nitrogen regulation.

Furthermore, microbial symbionts help seagrass cope with environmental stressors. For example, in marine sediments, sulfate-reducing prokaryotes perform the final step of the anaerobic food chain, which can produce toxic sulfide compounds (Smith et al. 2004). Although these compounds are a major reason for seagrass die-offs, microbial symbionts on healthy seagrasses are able to detoxify the environment by oxidizing these sulfides to sulfur or sulfate, contributing to the plant’s resilience. These further highlights how the health and survival of the seagrass plant itself depend on the functionality of its microbial partners.

## A changing interface under climate change

Seagrass meadows are increasingly at the forefront of climate change. Not only are seas warming, but marine heat waves are becoming ever more frequent and intense. Even in places such as northern Europe, which was thought only a decade ago to be buffered from marine heat waves, are now having multiple annual events. Within the intertidal areas, when marine heat waves are aligned with terrestrial heat waves, temperature spikes above 40 degrees Celsius have been recorded (Thomas et al. In review), and temperature increases are one of the probable drivers of negative seagrass trajectories globally (Turschwell et al. 2021).

Increasing temperatures and storminess have the capacity to alter many of the processes vital to seagrasses, potentially reducing the One Health benefits. However, their relative resilience to a changing climate means they’re well placed to resist heat if they don’t have other cumulative stressors (Unsworth et al. 2018a). This highlights the benefits of improving water quality and reducing other stressors in our coastal seas. Although it has not been studied in the context of seagrass, the ground cover of vegetation is well documented to reduce soil temperatures (Song et al. 2013). If such a concept applies to seagrass, this has considerable potential to buffer elevated temperature spikes from marine heat waves, potentially providing refuge for shallow-water biodiversity.

Such buffering capacity in the context of a changing climate extends to its critical role in shaping seawater carbonate chemistry through its influence on dissolved inorganic carbon dynamics (Unsworth et al. 2012). During photosynthesis, seagrasses uptake carbon dioxide, increasing local pH and carbonate ion concentration, which can enhance conditions for calcifying organisms such as shellfish and corals. Conversely, at night, respiration releases carbon dioxide, lowering pH and contributing to natural diel variability in carbonate chemistry. These processes can buffer coastal waters against ocean acidification, stabilizing local environments that support biodiversity and fisheries (Duarte 2017). However, the magnitude and persistence of this buffering effect depend on factors such as meadow density, hydrodynamics, light availability, and water residence time. Understanding these complex interactions is essential for accurately assessing seagrasses' role in coastal carbon cycling and their potential to mitigate the impacts of acidification.

## Seagrass and One Health in action

### The Adelaide example of seagrass and One Health

The case of seagrass loss and recovery off the metropolitan coast of Adelaide, Australia, provides a powerful example of the relationship between anthropogenic activity and ecosystem degradation in the context of One Health. For over half a century, the Adelaide coast was exposed to continuous discharges of nitrogenous waste from a variety of sources, including wastewater treatment plants and agricultural runoff (Tanner et al. 2014). This prolonged exposure to nutrient pollution resulted in the heavy loss of over 6200 hectares of seagrass, with significant societal consequences, with substantial implications for beach management, fisheries, and biodiversity (Tanner et al. 2014). The loss of these meadows directly affected the provisioning of ecosystem services that supported local livelihoods, such as commercial fisheries for King George whiting and blue swimmer crab (Jenkins et al. 2011).

However, the narrative does not end there. Following the implementation of water quality management plans to reduce nitrogen loads, a considerable recovery of seagrasses has occurred since the early 2000s, with a net recovery of 11,000 hectares in deeper waters (Fernandes et al. 2022). This example demonstrates a direct causal chain from pollution to degradation and from targeted policy to recovery. It shows that investing in environmental health, specifically by addressing the source of nutrient pollution, is a direct investment in a community’s long-term social and economic well-being. The Adelaide experience highlights that environmental policy is not separate from public health and economic policy but is, in fact, an essential component of a One Health approach.

### One Health as a potential framework for seagrass conservation policy

Effective seagrass conservation requires a shift from reactive, expensive restoration to proactive, multisectoral protection that includes restoration. This principle is central to the 10 Golden Rules for seagrass restoration, which emphasize that it is far more effective to protect existing meadows than to attempt to just replant them alone (Unsworth et al. 2025). This is particularly salient given that only 26% of known seagrass meadows fall within marine protected areas, compared with over 40% for coral reefs and mangroves (UNEP 2020). Within the majority of these areas, threats to those seagrasses remain prevalent (Jones et al. 2025). The challenges faced in restoration, such as the high cost and difficulty of establishing new meadows in high-energy environments, further underscore the need for a protective approach.

Successful conservation efforts must be collaborative, engaging a diverse range of stakeholders from local communities to government agencies (Ison et al. 2021). The Long Island Nitrogen Action Plan in the United States, implemented by agencies and local communities, was designed to reduce nitrogen pollution and improve water quality. Between 2016 and 2025, this action plan led to nitrogen loads from the watershed decreasing by 58.5% (Duvall and Hagy 2025). The plan’s success in addressing the leading threat to seagrass health in the region demonstrates that coordinated action can reverse the trend of degradation and the resultant wider ecosystem benefits that strongly align with a holistic One Health vision. Engaging local communities is not merely a formality but a necessity, because these groups often become the long-term custodians of conservation sites, ensuring the sustainability of efforts (Unsworth et al. 2025). Indeed, those stakeholders reliant on seagrass ecosystems for livelihoods ask for more protection (Boucek et al. 2024).

## Conclusions

The summary presented here establishes seagrass meadows as a foundational and tangible concept within the One Health paradigm. The interconnectedness of these ecosystems is not merely a theoretical construct but a demonstrable reality. The role of the seagrass holobiont, from its resilient genetics to the antibiotic-producing microbes on its leaves, directly influences the health of marine animals and, by extension, the human communities that depend on them for food, livelihood, and well-being. The pervasive global decline of seagrass meadows is a direct consequence of anthropogenic pressures, particularly nutrient pollution from urban and agricultural runoff. This environmental degradation has measurable negative consequences on human health and local economies, as is evidenced by case studies such as the one in Adelaide. However, the success stories of recovery, prompted by effective policy and collaborative governance, demonstrate that this trend is not irreversible.

A future-focused One Health imperative must recognize that the fate of these submerged ecosystems is inextricably linked to our own. This requires a shift in perspective, moving away from viewing environmental conservation as a separate, isolated concern. Instead, policymakers, researchers, and communities must adopt an integrated approach that recognizes the deep causal links between land use, water quality, ecosystem integrity, and human and animal health. This means implementing policies that not only protect existing seagrass but also address the root causes of their decline, such as nutrient and sediment pollution at the source. It also means investing in interdisciplinary research that can accurately quantify the full economic and social value of these ecosystems, providing a stronger financial incentive for their protection. Ultimately, the conservation and restoration of seagrass meadows are not just an ecological goal; they are an essential investment in a healthier, more resilient future for people, animals, and the planet we share (figure 1).

**Figure 1.**
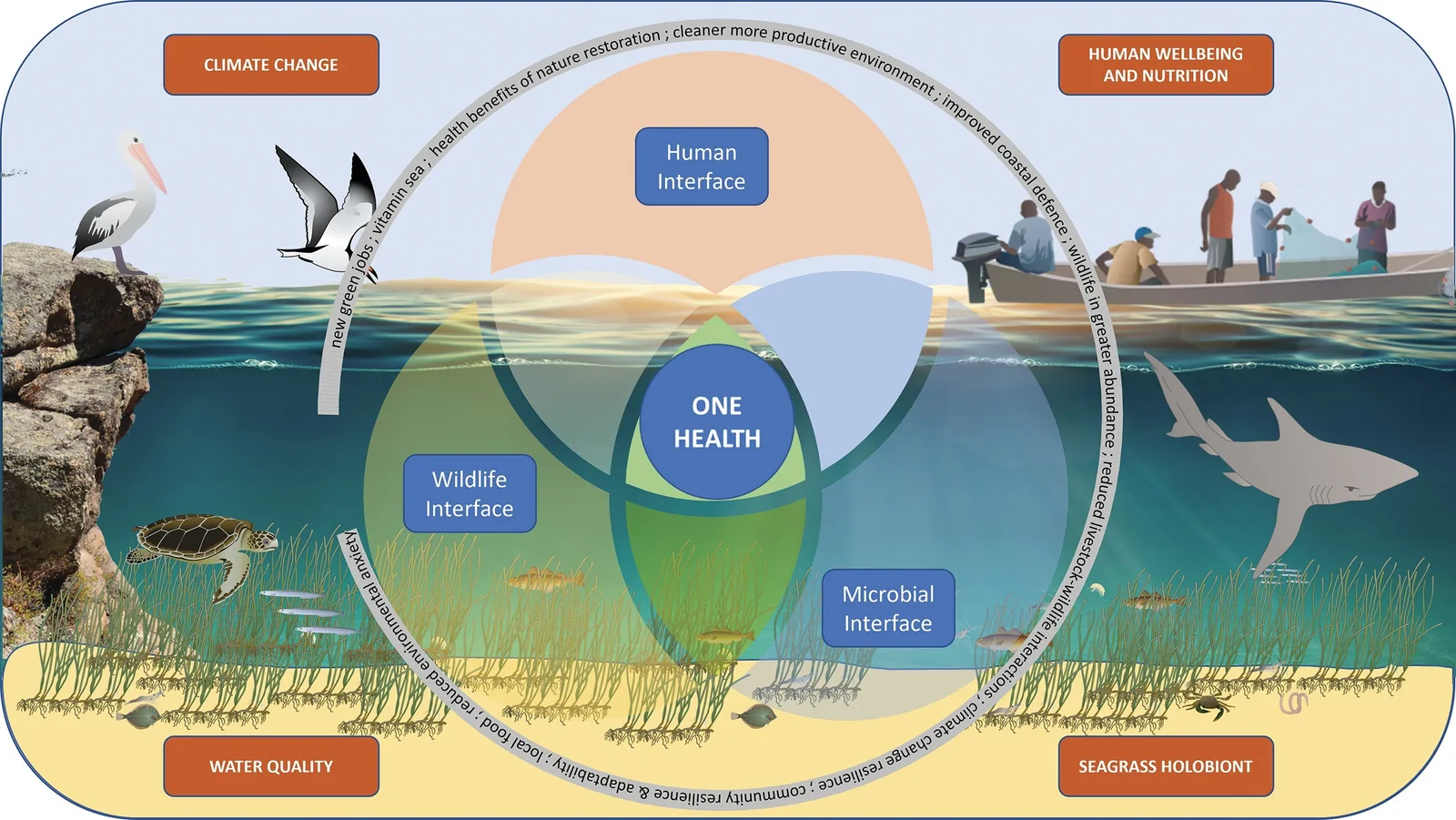
A conceptual framework of the three interlinked facets of seagrass as a foundational concept for One Health.
